# Hexaaqua­magnesium(II) bis­(d-camphor-10-sulfonate)

**DOI:** 10.1107/S1600536808018047

**Published:** 2008-06-21

**Authors:** Dejan Jeremić, Goran N. Kaluderović, Ilija Brčeski, Santiago Gómez-Ruiz, Katarina K. Andelković

**Affiliations:** a Faculty of Chemistry, University of Belgrade, Studentski trg 12–16, PO Box 158, 11000 Belgrade, Republic of Serbia; b Department of Chemistry, Institute of Chemistry, Technology and Metallurgy, Studentski trg 14, 11000 Belgrade, Republic of Serbia; c Departamento de Química Inorgánica y Analìtica, ESCET, Universidad Rey Juan Carlos, 28933 Móstoles, Madrid, Spain

## Abstract

The structure of the title complex, [Mg(H_2_O)_6_](C_10_H_15_O_4_S)_2_, consists of regular octa­hedral [Mg(H_2_O)_6_]^2+^ cations and d-camphor-10-sulfonate anions. A three-dimensional supra­molecular architecture is formed *via* hydrogen-bond inter­actions [O—H⋯O = 2.723 (2)–2.833 (2) Å] to give alternating layers of [Mg(H_2_O)_6_]^2+^ cations and d-camphor-10-sulfonate anions. The title compound is isomorphous with the zinc, copper, cadmium and nickel analogues.

## Related literature

For related literature, see: Baldacci (1938 ▶); Couldwell *et al.* (1978 ▶); Henderson & Nicholson (1995 ▶); Schepke *et al.* (2007 ▶); Zhou *et al.* (2003 ▶).
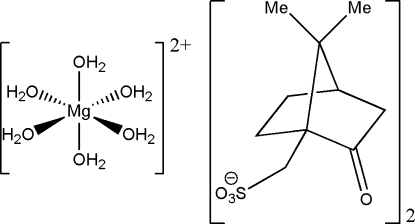


## Experimental

### 

#### Crystal data

[Mg(H_2_O)_6_](C_10_H_15_O_4_S)_2_
*M* *_r_* = 594.97
Monoclinic, 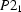

*a* = 11.75456 (10) Å
*b* = 7.05950 (8) Å
*c* = 17.22794 (15) Å
β = 93.1811 (8)°
*V* = 1427.39 (2) Å^3^
*Z* = 2
Mo *K*α radiation
μ = 0.27 mm^−1^
*T* = 130 (2) K
0.5 × 0.2 × 0.2 mm

#### Data collection

Oxford Diffraction Xcalibur CCD diffractometer
Absorption correction: multi-scan (*CrysAlis RED*; Oxford Diffraction, 2008 ▶) *T*_min_ = 0.917, *T*_max_ = 1.000 (expected range = 0.869–0.947)
40278 measured reflections
8136 independent reflections
7028 reflections with *I* > 2σ(*I*)
*R*_int_ = 0.025

#### Refinement

*R*[*F* ^2^ > 2σ(*F* ^2^)] = 0.028
*wR*(*F* ^2^) = 0.069
*S* = 0.99
8136 reflections
386 parameters
1 restraint
H atoms treated by a mixture of independent and constrained refinement
Δρ_max_ = 0.37 e Å^−3^
Δρ_min_ = −0.36 e Å^−3^
Absolute structure: Flack (1983 ▶), 3459 Friedel pairs
Flack parameter: 0.03 (4)

### 

Data collection: *CrysAlis CCD* (Oxford Diffraction, 2008 ▶); cell refinement: *CrysAlis RED* (Oxford Diffraction, 2008 ▶); data reduction: *CrysAlis RED*; program(s) used to solve structure: *SHELXS97* (Sheldrick, 2008 ▶); program(s) used to refine structure: *SHELXL97* (Sheldrick, 2008 ▶); molecular graphics: *ORTEP-3* (Farrugia, 1997 ▶); software used to prepare material for publication: *SHELXL97*.

## Supplementary Material

Crystal structure: contains datablocks I, global. DOI: 10.1107/S1600536808018047/pk2103sup1.cif

Structure factors: contains datablocks I. DOI: 10.1107/S1600536808018047/pk2103Isup2.hkl

Additional supplementary materials:  crystallographic information; 3D view; checkCIF report

## Figures and Tables

**Fig. 1.**
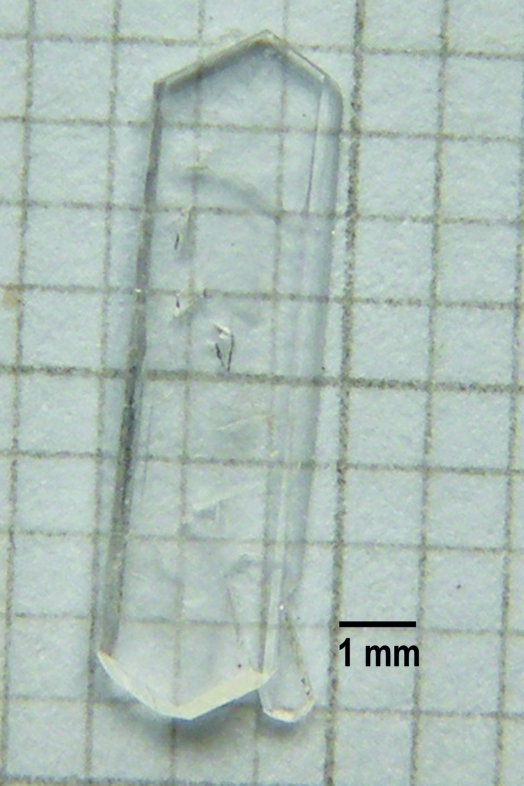
Crystals of [Mg(H2O)6](C10H15O4S)2.

**Fig. 2.**
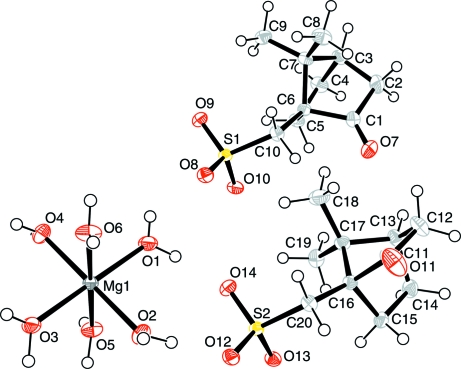
ORTEP representation of [Mg(H2O)6](C10H15O4S)2. Displacement ellipsoids are plotted at the 50% probability level and H atoms are shown as small spheres of arbitrary radius.

## supplementary crystallographic information

### Comment

The title compound was prepared by the reaction of magnesium tape with a solution containing D-camphor-10-sulfonic acid. Crystallization was achieved by placing a platinum wire into the solution to give a controlled zone cooling as well to provide nucleation centres. By this method we were able to produce crystals with dimensions typically from 4 × 1 × 0.4 mm to 12 × 10 × 3 mm (Fig. 1). Since crystals are easily obtainable in large size they could be of possible use as optical filters for UV/VIS/IR light, which is under further investigation as part of our ongoing research project. Also, the camphorsulfonate anion exhibits low toxicity (Baldacci, 1938) so its magnesium salt could be useful as a food supplement. The structure is isomorphous to those of analogous metal salts with Zn(II), Cu(II), Cd(II) and Ni(II), which have been structurally characterized previously (Couldwell *et al.*, 1978; Henderson & Nicholson, 1995; Schepke *et al.*, 2007; Zhou *et al.*, 2003).

As found in the crystal lattice of other isomorph compounds [*M*(H_2_O)_6_](C_10_H_15_O_4_S)_2_ (*M* = Zn(II), Cu(II), Cd(II) and Ni(II); Couldwell *et al.*, 1978; Henderson & Nicholson, 1995; Schepke *et al.*, 2007; Zhou *et al.*, 2003), the title compound structure consist of [Mg(H_2_O)_6_]^2+^ cations and two crystallogaphically independent D-camphor-10-sulfonate anions (Fig. 2). The magnesium atom in [Mg(H_2_O)_6_](C_10_H_15_O_4_S)_2_ has octahedral coordination. The Mg—O distances are in the range from 2.0315 (9) to 2.060 (2) Å and O—Mg—O angles are between 85.34 (6) and 94.23 (4)°. Extensive hydrogen-bonding stabilizes the structure (O···O distance = 2.723 (2)–2.833 (2) Å; O—H···O angle = 163 (3)–178 (2)°). These hydrogen bonds are formed between the coordinated water molecules and the O atoms of the SO_3_^-^ groups.

### Experimental

D-camphorsulfonic acid monohydrate (25.00 g) was dissolved in 80 ml of deionized water. Magnesium tape (2 g) was added and the solution was left at room temperature until all magnesium had dissolved. The solution was filtered, heated in a water bath and platinum wire (0.5 mm diameter, 10 cm long) was added as a crystallization centre. The solution was allowed too cool slowly in a water bath over the weekend. The monocrystals obtained were up to one centimetre in length, and were transparent in visible light. Crystals of a suitable size for X-ray analysis were also present.

### Refinement

The water H atoms were found and yielded reasonable bond lengths and angles (O—H bond length: 0.65 (3)–0.94 (3) Å), all other H atoms were positioned geometrically and treated as riding, with C—H bonding lengths constrained to 0.98–1.00 Å.

### Crystal data

**Table:**

[Mg(H_2_O)_6_](C_10_H_15_O_4_S)_2_	*F*_000_ = 636
*M**_r_* = 594.97	*D*_x_ = 1.384 Mg m^−^^3^
Monoclinic, *P*2_1_	Mo *K*α radiation λ = 0.71073 Å
Hall symbol: P 2yb	Cell parameters from 21884 reflections
*a* = 11.75456 (10) Å	θ = 2.9–32.2º
*b* = 7.05950 (8) Å	µ = 0.27 mm^−^^1^
*c* = 17.22794 (15) Å	*T* = 130 (2) K
β = 93.1811 (8)º	Prism, colourless
*V* = 1427.39 (2) Å^3^	0.5 × 0.2 × 0.2 mm
*Z* = 2	

### Data collection

**Table:**

Oxford Diffraction Xcalibur CCD diffractometer	8136 independent reflections
Monochromator: graphite	7028 reflections with *I* > 2σ(*I*)
Detector resolution: 16.356 pixels mm^-1^	*R*_int_ = 0.025
*T* = 130(2) K	θ_max_ = 30.5º
ω and φ scans	θ_min_ = 2.9º
Absorption correction: multi-scan(CrysAlis RED; Oxford Diffraction, 2008)	*h* = −16→16
*T*_min_ = 0.918, *T*_max_ = 1	*k* = −9→10
40278 measured reflections	*l* = −24→24

### Refinement

**Table:**

Refinement on *F*^2^	Hydrogen site location: inferred from neighbouring sites
Least-squares matrix: full	H atoms treated by a mixture of independent and constrained refinement
*R*[*F*^2^ > 2σ(*F*^2^)] = 0.028	*w* = 1/[σ^2^(*F*_o_^2^) + (0.0437*P*)^2^] where *P* = (*F*_o_^2^ + 2*F*_c_^2^)/3
*wR*(*F*^2^) = 0.069	(Δ/σ)_max_ = 0.001
*S* = 0.99	Δρ_max_ = 0.37 e Å^−^^3^
8136 reflections	Δρ_min_ = −0.36 e Å^−^^3^
386 parameters	Extinction correction: none
1 restraint	Absolute structure: Flack (1983), 3459 Friedel pairs
Primary atom site location: structure-invariant direct methods	Flack parameter: 0.03 (4)
Secondary atom site location: difference Fourier map	

### Special details

**Table:**

Experimental. CrysAlis RED: Empirical absorption correction using spherical harmonics, implemented in SCALE3 ABSPACK scaling algorithm.
Geometry. All e.s.d.'s (except the e.s.d. in the dihedral angle between two l.s. planes) are estimated using the full covariance matrix. The cell e.s.d.'s are taken into account individually in the estimation of e.s.d.'s in distances, angles and torsion angles; correlations between e.s.d.'s in cell parameters are only used when they are defined by crystal symmetry. An approximate (isotropic) treatment of cell e.s.d.'s is used for estimating e.s.d.'s involving l.s. planes.
Refinement. Refinement of *F*^2^ against ALL reflections. The weighted *R*-factor *wR* and goodness of fit *S* are based on *F*^2^, conventional *R*-factors *R* are based on *F*, with *F* set to zero for negative *F*^2^. The threshold expression of *F*^2^ > σ(*F*^2^) is used only for calculating *R*-factors(gt) *etc*. and is not relevant to the choice of reflections for refinement. *R*-factors based on *F*^2^ are statistically about twice as large as those based on *F*, and *R*- factors based on ALL data will be even larger.

### Fractional atomic coordinates and isotropic or equivalent isotropic displacement parameters (Å^2^)

**Table:**

	*x*	*y*	*z*	*U*_iso_*/*U*_eq_	
Mg1	0.25718 (3)	0.53807 (10)	0.005258 (19)	0.01645 (8)	
S1	0.91451 (2)	0.53891 (6)	0.154547 (14)	0.01601 (6)	
S2	0.42218 (2)	0.03871 (6)	0.169663 (14)	0.01538 (6)	
O1	0.39380 (8)	0.5349 (3)	0.08377 (6)	0.0314 (2)	
O2	0.17955 (11)	0.32080 (18)	0.06080 (7)	0.0233 (3)	
O3	0.11878 (8)	0.5400 (2)	−0.07079 (6)	0.0329 (2)	
O4	0.33376 (10)	0.75739 (18)	−0.05030 (7)	0.0230 (2)	
O5	0.33458 (11)	0.33056 (18)	−0.05722 (7)	0.0237 (3)	
O6	0.18050 (11)	0.74588 (18)	0.06692 (7)	0.0270 (3)	
O7	0.96709 (11)	0.29196 (18)	0.38660 (7)	0.0356 (3)	
O8	0.79319 (6)	0.5401 (2)	0.13121 (5)	0.02163 (17)	
O9	0.97210 (9)	0.71014 (15)	0.13082 (7)	0.0206 (2)	
O10	0.97185 (9)	0.36782 (15)	0.13013 (7)	0.0214 (2)	
O11	0.38282 (9)	0.10715 (19)	0.42956 (6)	0.0408 (3)	
O12	0.30410 (6)	0.03885 (19)	0.13849 (4)	0.02073 (16)	
O13	0.48094 (9)	−0.13891 (15)	0.15552 (6)	0.0196 (2)	
O14	0.48500 (9)	0.20329 (15)	0.14446 (7)	0.0210 (2)	
C1	1.03414 (13)	0.4160 (2)	0.37454 (8)	0.0252 (3)	
C2	1.13297 (14)	0.4805 (2)	0.42881 (9)	0.0325 (4)	
H2C	1.1889	0.3773	0.4389	0.039*	
H2D	1.1061	0.5268	0.4789	0.039*	
C3	1.18376 (12)	0.6419 (2)	0.38198 (8)	0.0242 (3)	
H3C	1.2325	0.7326	0.4136	0.029*	
C4	1.24354 (10)	0.5464 (3)	0.31519 (7)	0.0265 (3)	
H4C	1.2927	0.4407	0.3346	0.032*	
H4D	1.2904	0.6384	0.2876	0.032*	
C5	1.14265 (11)	0.4728 (2)	0.26156 (8)	0.0212 (3)	
H5C	1.1441	0.3329	0.2578	0.025*	
H5D	1.1451	0.5272	0.2087	0.025*	
C6	1.03495 (9)	0.5416 (3)	0.30208 (6)	0.0176 (2)	
C7	1.07629 (11)	0.7315 (2)	0.34042 (7)	0.0199 (3)	
C8	0.99319 (13)	0.8131 (3)	0.39685 (9)	0.0284 (3)	
H8A	1.0328	0.9063	0.4308	0.043*	
H8B	0.9639	0.7107	0.4285	0.043*	
H8C	0.9297	0.8746	0.3674	0.043*	
C9	1.10418 (12)	0.8870 (2)	0.28336 (8)	0.0248 (3)	
H9A	1.1558	0.8371	0.2456	0.037*	
H9B	1.1409	0.9929	0.3117	0.037*	
H9C	1.0338	0.9313	0.2561	0.037*	
C10	0.91934 (9)	0.5356 (3)	0.25743 (6)	0.0198 (2)	
H10A	0.8744	0.6449	0.2747	0.024*	
H10B	0.8797	0.4195	0.2735	0.024*	
C11	0.47837 (11)	0.0789 (2)	0.40963 (7)	0.0255 (3)	
C12	0.58803 (12)	0.0905 (2)	0.46036 (8)	0.0299 (4)	
H12A	0.5875	0.001	0.5046	0.036*	
H12B	0.6016	0.2205	0.4804	0.036*	
C13	0.67642 (10)	0.0338 (3)	0.40264 (6)	0.0242 (2)	
H13	0.7563	0.0716	0.4186	0.029*	
C14	0.66032 (12)	−0.1789 (2)	0.38768 (8)	0.0251 (3)	
H14A	0.6564	−0.2497	0.4371	0.03*	
H14B	0.7231	−0.2309	0.3581	0.03*	
C15	0.54503 (11)	−0.1880 (2)	0.33913 (8)	0.0208 (3)	
H15A	0.487	−0.2573	0.3675	0.025*	
H15B	0.5542	−0.2506	0.2884	0.025*	
C16	0.51133 (9)	0.0242 (2)	0.32789 (6)	0.0178 (2)	
C17	0.62946 (10)	0.1228 (2)	0.32492 (8)	0.0195 (3)	
C18	0.62088 (13)	0.3390 (2)	0.32693 (10)	0.0313 (3)	
H18A	0.5667	0.3768	0.3653	0.047*	
H18B	0.5946	0.3858	0.2755	0.047*	
H18C	0.6959	0.3929	0.3414	0.047*	
C19	0.70229 (10)	0.0660 (2)	0.25786 (7)	0.0237 (3)	
H19A	0.695	−0.0705	0.2486	0.036*	
H19B	0.7822	0.0973	0.2712	0.036*	
H19C	0.6763	0.1349	0.2108	0.036*	
C20	0.41039 (10)	0.0635 (2)	0.27123 (6)	0.0181 (3)	
H20A	0.3855	0.1951	0.2806	0.022*	
H20B	0.3476	−0.0204	0.2859	0.022*	
H1A	0.4206 (19)	0.618 (3)	0.1021 (13)	0.038 (7)*	
H1B	0.422 (2)	0.433 (4)	0.1065 (15)	0.053 (7)*	
H2A	0.224 (2)	0.225 (4)	0.0849 (14)	0.057 (7)*	
H2B	0.129 (2)	0.330 (4)	0.0758 (14)	0.043 (7)*	
H3A	0.084 (3)	0.436 (4)	−0.0930 (17)	0.078 (9)*	
H3B	0.0917 (15)	0.625 (3)	−0.0876 (11)	0.016 (5)*	
H4A	0.3031 (16)	0.840 (3)	−0.0697 (11)	0.024 (5)*	
H4B	0.3943 (18)	0.738 (3)	−0.0788 (12)	0.032 (5)*	
H5A	0.379 (2)	0.345 (4)	−0.0771 (14)	0.046 (8)*	
H5B	0.289 (2)	0.237 (4)	−0.0783 (15)	0.058 (7)*	
H6A	0.1136 (17)	0.735 (3)	0.0861 (11)	0.028 (5)*	
H6B	0.2102 (15)	0.842 (3)	0.0867 (10)	0.021 (5)*	

### Atomic displacement parameters (Å^2^)

**Table:**

	*U*^11^	*U*^22^	*U*^33^	*U*^12^	*U*^13^	*U*^23^
Mg1	0.01617 (16)	0.01541 (19)	0.01764 (16)	−0.0001 (2)	−0.00021 (12)	−0.0004 (2)
S1	0.01318 (10)	0.01544 (14)	0.01919 (11)	−0.00033 (17)	−0.00115 (8)	−0.00051 (18)
S2	0.01383 (11)	0.01487 (14)	0.01727 (11)	0.00042 (17)	−0.00063 (8)	−0.00043 (17)
O1	0.0348 (5)	0.0163 (5)	0.0407 (5)	−0.0004 (7)	−0.0202 (4)	−0.0014 (7)
O2	0.0194 (6)	0.0218 (7)	0.0290 (6)	0.0016 (5)	0.0047 (5)	0.0055 (5)
O3	0.0357 (5)	0.0154 (5)	0.0448 (5)	0.0009 (7)	−0.0222 (4)	0.0015 (7)
O4	0.0206 (6)	0.0205 (7)	0.0284 (6)	0.0041 (5)	0.0054 (5)	0.0046 (5)
O5	0.0222 (6)	0.0205 (7)	0.0293 (6)	−0.0030 (5)	0.0089 (5)	−0.0061 (5)
O6	0.0208 (6)	0.0243 (7)	0.0368 (7)	−0.0046 (5)	0.0091 (5)	−0.0110 (5)
O7	0.0463 (7)	0.0282 (7)	0.0319 (6)	−0.0108 (6)	−0.0003 (5)	0.0084 (5)
O8	0.0143 (3)	0.0222 (5)	0.0279 (4)	−0.0002 (6)	−0.0038 (3)	−0.0001 (6)
O9	0.0179 (5)	0.0171 (6)	0.0268 (5)	−0.0005 (4)	0.0010 (4)	0.0031 (4)
O10	0.0185 (5)	0.0189 (6)	0.0262 (5)	0.0010 (4)	−0.0024 (4)	−0.0050 (5)
O11	0.0286 (5)	0.0685 (10)	0.0260 (5)	0.0044 (5)	0.0075 (4)	−0.0109 (5)
O12	0.0154 (3)	0.0219 (5)	0.0242 (4)	−0.0005 (6)	−0.0048 (3)	0.0001 (6)
O13	0.0198 (5)	0.0178 (6)	0.0210 (5)	0.0023 (4)	−0.0013 (4)	−0.0032 (4)
O14	0.0198 (5)	0.0173 (6)	0.0259 (5)	−0.0012 (4)	0.0019 (4)	0.0040 (4)
C1	0.0293 (7)	0.0242 (8)	0.0216 (6)	0.0004 (6)	−0.0017 (5)	0.0019 (5)
C2	0.0360 (8)	0.0352 (10)	0.0252 (6)	−0.0009 (6)	−0.0085 (6)	0.0052 (6)
C3	0.0232 (6)	0.0257 (8)	0.0229 (6)	−0.0010 (6)	−0.0060 (5)	−0.0031 (5)
C4	0.0174 (5)	0.0306 (7)	0.0310 (6)	0.0049 (8)	−0.0046 (4)	−0.0059 (8)
C5	0.0176 (6)	0.0218 (7)	0.0239 (6)	0.0012 (5)	−0.0016 (5)	−0.0041 (5)
C6	0.0168 (4)	0.0174 (6)	0.0182 (4)	−0.0024 (7)	−0.0014 (3)	−0.0004 (7)
C7	0.0173 (6)	0.0210 (8)	0.0213 (6)	−0.0008 (5)	0.0010 (5)	−0.0042 (5)
C8	0.0253 (7)	0.0312 (9)	0.0293 (8)	−0.0015 (7)	0.0074 (6)	−0.0085 (7)
C9	0.0231 (6)	0.0207 (8)	0.0306 (7)	−0.0037 (6)	0.0010 (5)	−0.0004 (6)
C10	0.0154 (4)	0.0253 (6)	0.0187 (4)	−0.0024 (7)	0.0012 (4)	0.0009 (7)
C11	0.0255 (6)	0.0321 (10)	0.0192 (5)	0.0005 (6)	0.0027 (5)	−0.0044 (5)
C12	0.0301 (7)	0.0402 (11)	0.0189 (6)	0.0002 (6)	−0.0021 (5)	−0.0070 (5)
C13	0.0209 (5)	0.0309 (7)	0.0204 (5)	−0.0021 (8)	−0.0039 (4)	−0.0012 (8)
C14	0.0240 (6)	0.0263 (8)	0.0244 (6)	0.0026 (6)	−0.0034 (5)	0.0044 (6)
C15	0.0222 (6)	0.0188 (7)	0.0211 (6)	0.0003 (5)	−0.0011 (5)	0.0035 (5)
C16	0.0170 (5)	0.0194 (7)	0.0171 (4)	−0.0018 (6)	0.0000 (4)	0.0003 (6)
C17	0.0156 (6)	0.0195 (7)	0.0232 (6)	−0.0027 (5)	−0.0012 (4)	−0.0003 (5)
C18	0.0312 (7)	0.0220 (9)	0.0399 (8)	−0.0050 (6)	−0.0041 (6)	−0.0022 (6)
C19	0.0169 (5)	0.0306 (9)	0.0237 (5)	−0.0004 (6)	0.0021 (4)	0.0038 (6)
C20	0.0140 (4)	0.0215 (8)	0.0191 (5)	0.0014 (5)	0.0014 (4)	−0.0013 (5)

### Geometric parameters (Å, °)

**Table:**

Mg1—O3	2.0315 (9)	C5—H5C	0.99
Mg1—O1	2.0415 (10)	C5—H5D	0.99
Mg1—O2	2.0489 (14)	C6—C10	1.5245 (14)
Mg1—O6	2.0492 (14)	C6—C7	1.560 (2)
Mg1—O4	2.0541 (14)	C7—C9	1.522 (2)
Mg1—O5	2.0594 (14)	C7—C8	1.5286 (19)
S1—O9	1.4551 (12)	C8—H8A	0.98
S1—O10	1.4565 (11)	C8—H8B	0.98
S1—O8	1.4600 (7)	C8—H8C	0.98
S1—C10	1.7704 (11)	C9—H9A	0.98
S2—O14	1.4558 (11)	C9—H9B	0.98
S2—O13	1.4586 (11)	C9—H9C	0.98
S2—O12	1.4602 (7)	C10—H10A	0.99
S2—C20	1.7715 (11)	C10—H10B	0.99
O1—H1A	0.73 (2)	C11—C12	1.5190 (19)
O1—H1B	0.87 (3)	C11—C16	1.5308 (17)
O2—H2A	0.94 (3)	C12—C13	1.5309 (19)
O2—H2B	0.66 (2)	C12—H12A	0.99
O3—H3A	0.92 (3)	C12—H12B	0.99
O3—H3B	0.734 (19)	C13—C14	1.534 (3)
O4—H4A	0.75 (2)	C13—C17	1.5524 (18)
O4—H4B	0.90 (2)	C13—H13	1
O5—H5A	0.65 (3)	C14—C15	1.5538 (17)
O5—H5B	0.91 (3)	C14—H14A	0.99
O6—H6A	0.87 (2)	C14—H14B	0.99
O6—H6B	0.829 (19)	C15—C16	1.559 (2)
O7—C1	1.2038 (19)	C15—H15A	0.99
O11—C11	1.2092 (17)	C15—H15B	0.99
C1—C2	1.520 (2)	C16—C20	1.5195 (15)
C1—C6	1.5317 (19)	C16—C17	1.5568 (18)
C2—C3	1.536 (2)	C17—C19	1.5290 (18)
C2—H2C	0.99	C17—C18	1.530 (2)
C2—H2D	0.99	C18—H18A	0.98
C3—C4	1.5368 (19)	C18—H18B	0.98
C3—C7	1.5520 (19)	C18—H18C	0.98
C3—H3C	1	C19—H19A	0.98
C4—C5	1.5518 (17)	C19—H19B	0.98
C4—H4C	0.99	C19—H19C	0.98
C4—H4D	0.99	C20—H20A	0.99
C5—C6	1.5568 (17)	C20—H20B	0.99
			
O3—Mg1—O1	178.64 (5)	C8—C7—C3	113.12 (12)
O3—Mg1—O2	86.83 (6)	C9—C7—C6	114.83 (11)
O1—Mg1—O2	92.10 (6)	C8—C7—C6	113.46 (11)
O3—Mg1—O6	88.28 (6)	C3—C7—C6	94.10 (11)
O1—Mg1—O6	90.97 (6)	C7—C8—H8A	109.5
O2—Mg1—O6	94.23 (4)	C7—C8—H8B	109.5
O3—Mg1—O4	92.89 (6)	H8A—C8—H8B	109.5
O1—Mg1—O4	88.17 (6)	C7—C8—H8C	109.5
O2—Mg1—O4	179.49 (6)	H8A—C8—H8C	109.5
O6—Mg1—O4	85.34 (6)	H8B—C8—H8C	109.5
O3—Mg1—O5	91.65 (6)	C7—C9—H9A	109.5
O1—Mg1—O5	89.11 (6)	C7—C9—H9B	109.5
O2—Mg1—O5	86.14 (6)	H9A—C9—H9B	109.5
O6—Mg1—O5	179.62 (7)	C7—C9—H9C	109.5
O4—Mg1—O5	94.30 (4)	H9A—C9—H9C	109.5
O9—S1—O10	112.20 (5)	H9B—C9—H9C	109.5
O9—S1—O8	112.36 (7)	C6—C10—S1	118.88 (7)
O10—S1—O8	112.69 (7)	C6—C10—H10A	107.6
O9—S1—C10	107.62 (8)	S1—C10—H10A	107.6
O10—S1—C10	106.77 (8)	C6—C10—H10B	107.6
O8—S1—C10	104.62 (5)	S1—C10—H10B	107.6
O14—S2—O13	112.56 (5)	H10A—C10—H10B	107
O14—S2—O12	112.17 (7)	O11—C11—C12	126.85 (12)
O13—S2—O12	112.85 (7)	O11—C11—C16	126.12 (12)
O14—S2—C20	106.54 (7)	C12—C11—C16	107.04 (10)
O13—S2—C20	108.27 (7)	C11—C12—C13	101.36 (10)
O12—S2—C20	103.79 (5)	C11—C12—H12A	111.5
Mg1—O1—H1A	125.6 (18)	C13—C12—H12A	111.5
Mg1—O1—H1B	124.8 (16)	C11—C12—H12B	111.5
H1A—O1—H1B	108.9 (18)	C13—C12—H12B	111.5
Mg1—O2—H2A	119.9 (14)	H12A—C12—H12B	109.3
Mg1—O2—H2B	123 (2)	C12—C13—C14	106.47 (13)
H2A—O2—H2B	113 (3)	C12—C13—C17	103.46 (11)
Mg1—O3—H3A	126.0 (18)	C14—C13—C17	102.52 (10)
Mg1—O3—H3B	125.1 (14)	C12—C13—H13	114.4
H3A—O3—H3B	108.7 (18)	C14—C13—H13	114.4
Mg1—O4—H4A	125.3 (14)	C17—C13—H13	114.4
Mg1—O4—H4B	121.2 (13)	C13—C14—C15	103.09 (10)
H4A—O4—H4B	104.6 (19)	C13—C14—H14A	111.1
Mg1—O5—H5A	124 (2)	C15—C14—H14A	111.1
Mg1—O5—H5B	117.1 (15)	C13—C14—H14B	111.1
H5A—O5—H5B	112 (3)	C15—C14—H14B	111.1
Mg1—O6—H6A	124.5 (13)	H14A—C14—H14B	109.1
Mg1—O6—H6B	127.8 (12)	C14—C15—C16	103.62 (11)
H6A—O6—H6B	106.4 (17)	C14—C15—H15A	111
O7—C1—C2	126.67 (13)	C16—C15—H15A	111
O7—C1—C6	126.55 (13)	C14—C15—H15B	111
C2—C1—C6	106.77 (12)	C16—C15—H15B	111
C1—C2—C3	101.85 (11)	H15A—C15—H15B	109
C1—C2—H2C	111.4	C20—C16—C11	108.45 (10)
C3—C2—H2C	111.4	C20—C16—C17	124.28 (12)
C1—C2—H2D	111.4	C11—C16—C17	100.90 (10)
C3—C2—H2D	111.4	C20—C16—C15	116.06 (12)
H2C—C2—H2D	109.3	C11—C16—C15	101.75 (11)
C2—C3—C4	105.97 (13)	C17—C16—C15	102.29 (10)
C2—C3—C7	102.39 (11)	C19—C17—C18	108.60 (13)
C4—C3—C7	103.17 (10)	C19—C17—C13	111.01 (11)
C2—C3—H3C	114.6	C18—C17—C13	113.88 (13)
C4—C3—H3C	114.6	C19—C17—C16	116.45 (11)
C7—C3—H3C	114.6	C18—C17—C16	112.67 (12)
C3—C4—C5	103.09 (10)	C13—C17—C16	93.77 (10)
C3—C4—H4C	111.1	C17—C18—H18A	109.5
C5—C4—H4C	111.1	C17—C18—H18B	109.5
C3—C4—H4D	111.1	H18A—C18—H18B	109.5
C5—C4—H4D	111.1	C17—C18—H18C	109.5
H4C—C4—H4D	109.1	H18A—C18—H18C	109.5
C4—C5—C6	104.03 (10)	H18B—C18—H18C	109.5
C4—C5—H5C	111	C17—C19—H19A	109.5
C6—C5—H5C	111	C17—C19—H19B	109.5
C4—C5—H5D	111	H19A—C19—H19B	109.5
C6—C5—H5D	111	C17—C19—H19C	109.5
H5C—C5—H5D	109	H19A—C19—H19C	109.5
C10—C6—C1	110.45 (11)	H19B—C19—H19C	109.5
C10—C6—C5	119.30 (10)	C16—C20—S2	121.03 (8)
C1—C6—C5	103.12 (12)	C16—C20—H20A	107.1
C10—C6—C7	119.05 (14)	S2—C20—H20A	107.1
C1—C6—C7	99.69 (10)	C16—C20—H20B	107.1
C5—C6—C7	102.46 (10)	S2—C20—H20B	107.1
C9—C7—C8	107.88 (13)	H20A—C20—H20B	106.8
C9—C7—C3	113.15 (11)		

### Hydrogen-bond geometry (Å, °)

**Table:**

*D*—H···*A*	*D*—H	H···*A*	*D*···*A*	*D*—H···*A*
O5—H5OA···O6^i^	0.89 (3)	1.87 (3)	2.7329 (18)	163 (3)
O1—H1A···O13^ii^	0.73 (2)	2.06 (2)	2.782 (2)	175 (2)
O1—H1B···O14	0.88 (3)	1.89 (3)	2.757 (2)	174 (2)
O2—H2A···O12	0.94 (3)	1.84 (3)	2.7711 (17)	176 (2)
O2—H2B···O10^iii^	0.66 (2)	2.13 (2)	2.7966 (17)	176 (2)
O3—H3A···O9^iv^	0.91 (3)	1.83 (3)	2.7406 (17)	173 (3)
O3—H3B···O10^v^	0.73 (2)	1.99 (2)	2.7230 (17)	175.6 (18)
O4—H4A···O8^v^	0.75 (2)	2.07 (2)	2.8146 (17)	173 (2)
O4—H4B···O14^v^	0.90 (2)	1.88 (2)	2.7748 (16)	176.1 (19)
O5—H5A···O13^v^	0.65 (2)	2.19 (2)	2.8327 (16)	172 (3)
O5—H5B···O8^iv^	0.91 (3)	1.90 (3)	2.8058 (17)	173 (2)
O6—H6A···O9^iii^	0.87 (2)	1.88 (2)	2.7524 (17)	178 (2)
O6—H6B···O12^ii^	0.83 (2)	1.959 (19)	2.7767 (17)	169.5 (18)
C10—H10B···O7	0.99	2.33	2.844 (2)	111

Symmetry codes: (i) +1, +2, +1; (ii) *x*, *y*+1, *z*; (iii) *x*−1, *y*, *z*; (iv) −*x*+1, *y*−1/2, −*z*; (v) −*x*+1, *y*+1/2, −*z*.
